# Variety in Diet

**Published:** 1876-08

**Authors:** Alton Cheswicke


### VARIETY IN DIET,

### IN ITS RELATIONS TO HEALTH.

By Alton Cheswicke.

Variety has been justly termed "the spice of life." We think we may go even further, and claim for it that it is one of the *essentials* of healthful, vigorous life. Man's needs are so many and so diversified, and it seems to have been ordained of nature that they should be supplied through so many different channels, and from so many different sources—no one of which is sufficient of itself to answer all his requirements—that if voluntarily, or through force of circumstances, he confines himself, or is confined to any one of them alone for the needed ministrations to his necessities, he is certain in some part of his organism, more or less important, to go famished for want of what this alone can not afford him. This is essentially true of all departments of his being; but is, perhaps, more readily perceptible in his physical system, whose workings come more directly within the range of our observations. And in none of the various ways in which the physical system is built up and sustained is this element of variety seen to be more essential than in the matter of diet. The man whose dietary is most extended in its range—who is least restricted to, *and dependent upon*, any particular article or articles of food—will possess the most perfect health in every respect. It may be urged here that any very extensive variety in food is possible only to those who have extensive means at their command ; and we may further be pointed to the fact that the wealthy man, whose table is loaded at every meal with the endless variety which the exhaustless resources of civilization can afford him at the bidding of wealth, is less likely, for all this, to possess vigorous health than the laborer who breakfasts, dines, and sups, week in and week out, on crackers and red herring, bread and cheese, or any such simple and primitive bill of fare. To this, however, we would only reply that indiscriminate and unwholesome combinations of different articles of food is very different from wholesome variety, and that this wholesome variety is within the reach—if not (as we are inclined to think) of every individual—at least, of the great majority of mankind ; and it is with the majority that we have now to do.

To secure starting aright, we will first of all refer the subject to nature, and calling in analogy to our aid, will begin by likening man to the turkey—a comparison, by the way, that would be far less complimentary to the former in French than it is in English. As regards the character of his food, the turkey, or more comprehensively speaking, the whole gallinaceous tribe of which he is the type, may be justly considered as the prototype of man among the feathered race, holding, as he does, about the same relative position to the strictly flesh-eating and grain-eating birds, that man does to the carnivorous and graminivorous quadrupeds. The turkey is essentially omnivorous ; his bill of fare comprising, in his native state of freedom, not only grasses, grains, seeds, berries, acorns, nuts, and an endless number and variety of grubs and insects, but also slugs, snails, worms, and small reptiles, such as tadpoles, lizards, young frogs, etc. ; nor does he disdain, by way of an occasional relish, to regale himself, now and then, upon moles, field-mice, and other similar small fry among the warm-blooded tenants of the woods and fields. Thus, we perceive, his dietary proves him to be at once graminivorous, granivorous, frugivorous, insectivorous, and, in a mild way, carnivorous, since he dines occasionally off the flesh of creatures both above and below him in the scale of development—in a word, thoroughly *omnivorous*—as is man. Moreover, while the race of gallinaceous birds are, like man, unprovided with natural weapons to enable them to destroy any living creature not vastly smaller than themselves, they yet, like him, have a decided relish for flesh diet, when it is brought within their reach, as any housewife can testify who has fed scraps of cooked meat to poultry, and seen with what avidity and seeming advantage it is devoured, or who has detected them, in default of such diet, making a meal off their own or each other's eggs.

☛ Now, having shown the strong resemblance that exists between man and the turkey, as regards the character of their respective diet, we come to the natural conclusion that the two must, in respect to the point in which they are allied, be subject to similar laws—that what is good for the turkey in this respect, must, in a greater or less degree, be good for the man also. We think a little investigation will show that this is the lesson intended to be taught by nature—who is never mistaken—by this analogy. Let us see.

It is generally conceded that the flesh of the wild turkey is vastly superior, both in delicacy of flavor and nutritious quality, to that of the same animal in a state of domestication. This fact needs no explanation, when we consider that the character of the flesh of any animal depends so entirely upon the character and quality of the food that goes to form it, and can only attain its maximum quality when the food is most perfectly adapted to the needs of the system ; and that the appetite, when unperverted, is a certain and sure indication of what those needs are. The turkey's naturally extensive bill of fare comprises an immense variety in each and all of the different items enumerated ; and in a wild state it is all available for him at all times. Firstly, because Nature spreads a bounteous table for him, not one item being omitted or forgotten, and secondly, because, as no one article of food is to be found exclusively by itself in any great quantity—grains, insects, and reptiles being always found together, or, at all events, never very far apart, as they are mutually dependent on one another for their subsistance—and the whole being spread over a wide area, over which he must roam, taking what comes along, he is in no danger of being restricted long to any one item, and at the same time is certain in the wholesome and never-ending variety to be able to obtain just what he wants *at the time he wants it*—a very important provision indeed. Consequently, his health is perfect, and his flesh can not be otherwise. In a state of domestication, which is usually a state of comparative confinement—always so at the fattening period—he is no longer free to choose and select for himself ; and his diet, depending solely upon his keepers, is very much restricted, being usually limited to two or three articles of food at the most ; consequently, as there is no one article of food that can supply him everything, some of the wants of his system must go unprovided for. That such is the case, no one will question who has witnessed the frantic eagerness with which a lot of fowls that have been cooped up for any length of time, and fed exclusively on corn, their favorite dish to all appearance, will rush to obtain a few spears of freshly plucked green grass that may be thrown to them.

[TO BE CONTINUED.

